# Engineered exosomes for breast cancer immunotherapy: Emerging strategies and translational potential

**DOI:** 10.1016/j.bbrep.2026.102822

**Published:** 2026-10-01

**Authors:** Niloufar Orooji, Mahsa Ghasemian-Irani, Nasrin Salari, Tamana Eskandari, Mehrshad Shams, Fatemeh Tavassoli Razavi, Dariush Haghmorad

**Affiliations:** a Cancer Research Center, Semnan University of Medical Sciences, Semnan, Iran; b Department of Immunology, School of Medicine, Semnan University of Medical Sciences, Semnan, Iran; c Department of Immunology, Faculty of Medicine, Tabriz University of Medical Sciences, Tabriz, Iran; d Immunology Research Center, Tabriz University of Medical Sciences, Tabriz, Iran; e Department of Immunology, School of Medicine, Mazandaran University of Medical Sciences, Sari, Iran

**Keywords:** Engineered exosomes, Breast cancer, Immunotherapy, Targeted drug delivery, Nanocarriers, Extracellular vesicles

## Abstract

Breast cancer remains one of the leading causes of cancer-related mortality in women worldwide, despite significant advances in early detection and conventional therapies. Immunotherapy has emerged as a promising strategy to harness the body's immune system to combat tumors. However, limitations such as immune escape mechanisms and inefficient drug delivery continue to challenge its effectiveness. In recent years, engineered exosomes, cell-derived nanoscale vesicles, have gained attention as innovative carriers for cancer immunotherapy due to their natural biocompatibility, low immunogenicity, and inherent targeting capabilities. This review highlights recent advancements in the design and functionalization of exosomes for breast cancer immunotherapy, including the loading of immune-stimulating molecules, genetic cargos, and surface modifications to enhance tumor specificity. We also discuss their advantages over conventional nanocarriers, immunological mechanisms, preclinical evidence, and challenges related to clinical translation. While significant progress has been made, further research is needed to overcome current barriers and fully exploit the potential of engineered exosomes as a next-generation platform in breast cancer immunotherapy.

## Highlights

• Engineered exosomes improve targeted breast cancer immunotherapy.
• Engineered exosomes enhance immune activation and tumor targeting in breast cancer.
• Exosome vaccines stimulate adaptive and innate antitumor immunity.
• Engineered exosomes efficiently deliver therapeutic cargos to breast tumors.
• Clinical translation requires scalable and standardized exosome production.

## 1 Introduction

Breast cancer is the most frequently diagnosed malignancy among women and remains a major cause of cancer-related mortality worldwide. As its global prevalence continues to rise, understanding the biological heterogeneity of breast cancer is critical for developing effective and individualized therapeutic strategies [1,2]. According to the Global Burden of Disease (GBD) 2021 study, breast cancer accounted for the highest number of disability-adjusted life years (DALYs) among women across all age groups, with mortality second only to lung cancer. Breast cancer-related DALYs are projected to increase from 238.6 (95% CI: 223.2–254.2) per 100,000 in 2022 to 239.5 (95% CI: 186.9–300.6) per 100,000 b y 2050 [3].

Approximately 30% of global breast cancer cases are attributable to modifiable risk factors such as obesity, physical inactivity, and alcohol consumption, indicating a significant opportunity for prevention [4,5]. In addition, various reproductive and hormonal factors -including early menarche, late menopause, delayed childbirth, low parity, insufficient breastfeeding, use of hormone replacement therapy or oral contraceptives, and increased breast density-have been linked to elevated breast cancer risk and contribute to the global disease burden [6,7].

Among the molecular subtypes of breast cancer, triple-negative breast cancer is the most aggressive, characterized by the absence of estrogen receptor (ER), progesterone receptor (PR), and human epidermal growth factor receptor 2 (HER2). Patients with metastatic triple-negative breast cancer face a poor prognosis, with a five-year survival rate below 30%, underscoring the urgent need for novel and effective treatment options [8]. For patients with advanced or metastatic breast cancer, particularly those with aggressive subtypes, it is imperative to expand therapeutic approaches that improve clinical outcomes while reducing side effects, treatment costs, and the impact on quality of life [9].

Recent years have seen a shift in breast cancer treatment paradigms with the introduction of targeted therapies and immunotherapies that exploit specific genetic and immunological features of tumors [10]. Targeted agents such as trastuzumab and antibody–drug conjugates (ADCs) have significantly improved outcomes in HER2-positive breast cancer, supporting the development of more personalized treatment approaches. Immunotherapy strategies, including immune checkpoint inhibitors and cancer vaccines, have shown promise, particularly in breast cancer subtypes that lack hormone receptor expression and have fewer treatment options. These findings support the idea that integrating immunotherapy with molecularly targeted agents may help meet unmet clinical needs and improve patient outcomes [11].

Numerous preclinical and clinical investigations have evaluated the efficacy of immunotherapeutic approaches in breast cancer. Immune checkpoint inhibitors have been tested both as monotherapies and in combination with other modalities such as chemotherapy, monoclonal antibodies, antibody–drug conjugates, and cyclin-dependent kinase 4/6 (CDK4/6) inhibitors. Several of these combinations have been approved by the FDA following encouraging results from clinical trials. However, other studies have produced inconsistent findings or raised safety concerns that led to early termination. These mixed results underscore the complexity of the tumor microenvironment and the need to better understand its role in immunotherapy responses. Consequently, increasing attention is being given to the identification of reliable biomarkers that can guide patient selection, enhance therapeutic efficacy, and minimize immune-related adverse events [[12], [13], [14]]. Despite the clinical promise of immunotherapy, significant challenges remain. Many immunotherapeutic agents, such as cytokines, exhibit short systemic half-lives and poor tumor tissue penetration, often requiring high doses that are associated with toxicity, immune suppression, and potential immunogenicity [2].

In parallel, advances in nanomedicine have introduced a variety of nanoparticle-based delivery systems and phytochemical carriers aimed at improving drug delivery and bioavailability. While these strategies offer improved solubility and targeted delivery, their clinical translation is hindered by issues related to safety, toxicity, in vivo stability, and interactions with biological barriers [15,16]. Nanoparticles may be recognized and cleared by the immune system, accumulate in non-target organs, or be rapidly removed by hepatic and renal filtration, all of which compromise therapeutic efficacy and raise safety concerns [17,18].

To address these limitations, extracellular vesicles (EVs), including exosome-based systems, have emerged as promising nanocarriers in cancer immunotherapy. EVs are naturally secreted membrane-enclosed particles that can transport proteins, nucleic acids, and lipids and may possess favorable biocompatibility and cellular interaction properties. In this review, the term “EVs” is used as the broader designation, whereas “exosomes” is retained when referring to studies or engineered formulations that specifically use this term or provide evidence consistent with an endosomal origin. Engineered exosomes have been developed to disrupt immune checkpoints such as the CD47–SIRPα axis, thereby enhancing macrophage-mediated tumor clearance with fewer side effects compared to monoclonal antibodies [19,20].

Moreover, dendritic cell-derived EVs (DC-derived EVs) are being explored as antigen-presenting platforms in cancer vaccines, capable of stimulating effective T cell responses. Tumor-derived EVs (TEXs), although often associated with immunosuppression, can also be modified to carry tumor-associated antigens and induce strong anti-tumor immunity. Nonetheless, concerns regarding the oncogenic potential of their native cargo require careful evaluation [21,22].

Although EV- and exosome-based therapies show considerable potential, their clinical translation faces hurdles such as large-scale manufacturing, standardization of isolation and loading methods, and regulatory approval. Continued research is needed to optimize EV engineering and exosome-based therapeutic platforms, improve targeting specificity, and integrate these platforms with existing immunotherapies to achieve safe and effective clinical outcomes.

In this review, we summarize current advances in the application of engineered exosomes for breast cancer immunotherapy, with a focus on their design, immunological mechanisms, therapeutic potential, and challenges in clinical translation.

## 2 Exosome biology and breast cancer-specific mechanisms of action

### 2.1 Exosome biogenesis and molecular characteristics

Extracellular vesicles (EVs) are lipid bilayer-enclosed particles released by cells that mediate intercellular communication through the transfer of proteins, nucleic acids, and lipids to recipient cells. According to the updated MISEV2023 recommendations, EV is the preferred general term for cell-derived lipid bilayer-enclosed particles, whereas the term “exosome” should be used cautiously because it refers specifically to vesicles of endosomal origin that require experimental confirmation. When the biogenesis pathway cannot be conclusively demonstrated, operational terms such as small extracellular vesicles (sEVs) are considered more appropriate [23]. In this review, the term “exosome” is retained when referring to published studies that use this terminology or when discussing engineered platforms historically described as exosomes; however, EV and sEV are used where the available evidence does not establish a specific biogenesis pathway.

Exosomes are nanoscale EVs that originate from the endosomal pathway and are released by various cell types. Their biogenesis begins with inward budding of the plasma membrane, resulting in the formation of early endosomes, which subsequently mature into late endosomes and multivesicular bodies (MVBs). During MVB maturation, inward budding of the endosomal membrane generates intraluminal vesicles (ILVs). Following fusion of MVBs with the plasma membrane, ILVs are released into the extracellular environment as EVs. Although MVBs represent larger intracellular compartments, the released vesicles maintain their characteristic nanoscale dimensions, generally ranging from approximately 40–150 nm [24,25].

Two major molecular pathways contribute to EV biogenesis and cargo sorting: ESCRT-dependent and ESCRT-independent mechanisms. The endosomal sorting complex required for transport (ESCRT) machinery, including ESCRT-0, ESCRT-I, ESCRT-II, and ESCRT-III complexes, regulates membrane remodeling, ILV formation, and selective cargo incorporation. In contrast, ESCRT-independent pathways involve lipid domains and membrane-associated molecules, including tetraspanins such as CD9, CD63, CD81, and CD82, as well as heat shock proteins (HSP60, HSP70, and HSP90), which contribute to vesicle formation and molecular cargo selection [26,27].

EV cargo composition reflects the molecular characteristics and physiological status of donor cells. Proteomic analyses have identified both common EV-associated proteins and cell-specific molecules, including signaling proteins, transcription factors, enzymes, and extracellular matrix-related components [28]. In addition to proteins, EVs contain multiple classes of nucleic acids, including mRNAs, microRNAs, and DNA fragments, which can modify gene expression and cellular behavior in recipient cells. Lipid components such as cholesterol, sphingomyelin, and lysobisphosphatidic acid (LBPA) contribute to vesicle stability, membrane organization, and ILV formation within MVBs [29]. Through the delivery of these bioactive cargos, EVs can regulate signaling pathways, alter cellular phenotypes, and influence pathological processes, including cancer progression and immune modulation [30].

In breast cancer, these intrinsic biological properties provide the basis for tumor–stromal and tumor–immune communication. Tumor cells exploit EV-mediated transfer mechanisms to remodel the tumor microenvironment (TME), whereas immune cell-derived EVs can be engineered or utilized as therapeutic platforms to enhance antitumor immunity. Therefore, understanding the mechanisms governing EV formation, cargo selection, and recipient-cell interaction is essential for developing effective engineered exosome-based immunotherapies.

### 2.2 Breast cancer-specific mechanisms of EV uptake and cellular communication

Recipient-cell uptake represents a critical determinant of EV-mediated biological effects. EV internalization occurs through multiple mechanisms, including clathrin-dependent endocytosis, caveolae-mediated uptake, macropinocytosis, phagocytosis, and direct membrane fusion. The relative contribution of each pathway depends on recipient-cell phenotype, surface receptor expression, EV membrane composition, and cellular state [31,32].

Although these mechanisms have been described across different biological systems, breast cancer studies demonstrate that EV uptake is highly dependent on tumor-cell characteristics and specific molecular interactions rather than occurring through a universal pathway.

Tran et al. investigated an engineered EV-based delivery system in breast cancer models by modifying HEK293F-derived exosomes with tumor-penetrating peptides iRGD and tLyp1. These peptides were incorporated individually or in combination to enhance tumor targeting. Doxorubicin-loaded dual-targeted exosomes demonstrated enhanced uptake in both MCF-7 and MDA-MB-231 breast cancer cells compared with non-targeted or single-ligand formulations. Furthermore, dual-targeted exosomal doxorubicin exhibited markedly improved cytotoxic activity, with IC_50_/GI_50_ values 3.7–17.0-fold lower than free doxorubicin and other exosomal formulations [33]. These findings provide direct evidence that modification of EV surface properties can enhance breast cancer-cell recognition, intracellular delivery, and therapeutic activity.

Fuentes et al. further elucidated the molecular mechanisms regulating EV uptake in breast cancer. Using MDA-MB-231 cells, they demonstrated that sEV internalization was mediated, at least partly, by interactions between integrin β3 (ITGB3) and cell-surface heparan sulfate proteoglycans (HSPGs). Disruption of ITGB3 or HSPGs significantly reduced EV uptake, while inhibition of dynamin-dependent endocytosis impaired vesicle internalization. Mechanistically, ITGB3-associated focal adhesion kinase (FAK) activation was implicated in this process, identifying the ITGB3–HSPG–FAK axis as an important regulator of EV capture by aggressive breast cancer cells [34].

The recipient-cell phenotype also influences EV uptake efficiency. Stranford et al. compared EV internalization patterns in different breast cancer cell models, including MCF-7 and MDA-MB-231 cells, and demonstrated differences in uptake efficiency between these phenotypically distinct cell lines [35]. The higher uptake observed in the more aggressive MDA-MB-231 model highlights the importance of tumor subtype and cellular context in determining EV delivery efficiency. These findings indicate that successful EV-based therapeutic strategies should consider not only vesicle engineering but also the biological characteristics of the target tumor population.

The dynamic release and uptake of EVs further contribute to their complex biological functions. Cells continuously exchange EVs, generating heterogeneous vesicle populations with diverse molecular properties. Although studies in non-breast cancer models have provided important mechanistic insights into EV biology, their relevance to breast cancer should be interpreted cautiously. For example, genetically engineered mouse models have demonstrated that EV-mediated horizontal transfer of biological information can occur under inflammatory conditions and may be enhanced by immune activation or chemotherapy [23,36]. Similarly, studies in glioblastoma and neural stem cell models have shown that cellular stress and transformation can modify EV cargo composition and functional activity [37,38]. These observations provide general mechanistic principles of EV biology but require validation in breast cancer-specific systems.

Collectively, EV biogenesis, molecular composition, and uptake mechanisms determine their biological effects within the tumor microenvironment. Breast cancer-specific studies demonstrate that EV internalization is governed by a combination of tumor-cell phenotype, receptor expression, and vesicle surface characteristics. These findings provide the biological rationale for engineering EVs and exosome-based platforms with improved targeting specificity, enhanced intracellular delivery, and optimized therapeutic performance in breast cancer immunotherapy.

### 2.3 Functional roles of tumor-derived and immune cell-derived exosomes in breast cancer

Beyond their fundamental biological functions, EVs serve as important mediators of communication within the breast cancer TME. Depending on their cellular origin and molecular cargo, EVs can exert either tumor-promoting or tumor-suppressive effects. In breast cancer, TEXs primarily contribute to disease progression by promoting immune evasion, angiogenesis, and metastatic dissemination, whereas IEXs possess immunostimulatory properties and provide a biological foundation for the development of engineered EV-based immunotherapies. Understanding these opposing functions is essential for designing therapeutic strategies that selectively suppress tumor-promoting EV activity while exploiting immune-active EV platforms.

#### 2.3.1 Tumor-derived exosomes and breast cancer progression

Tumor-derived EVs represent a major component of breast cancer intercellular communication. These vesicles contain diverse bioactive molecules, including proteins, integrins, microRNAs, long non-coding RNAs, and growth factors, which can modify the behavior of tumor cells and surrounding stromal and immune populations [39,40]. Through horizontal transfer of these cargos, TEXs contribute to remodeling of the tumor microenvironment and facilitate processes associated with tumor growth, invasion, and metastatic progression.

One of the important roles of TEXs in breast cancer is the establishment of a supportive metastatic niche. TEX-associated integrins and signaling molecules can influence interactions between tumor cells and distant tissues, thereby facilitating organ-specific metastatic colonization [41,42]. In breast cancer, a notable example is the transfer of miR-105 through TEXs, which targets the tight junction protein zonula occludens-1 (ZO-1). This interaction disrupts endothelial barrier integrity, increases vascular permeability, and promotes tumor cell dissemination [43,44] (Fig. 1).

**Fig. 1:**
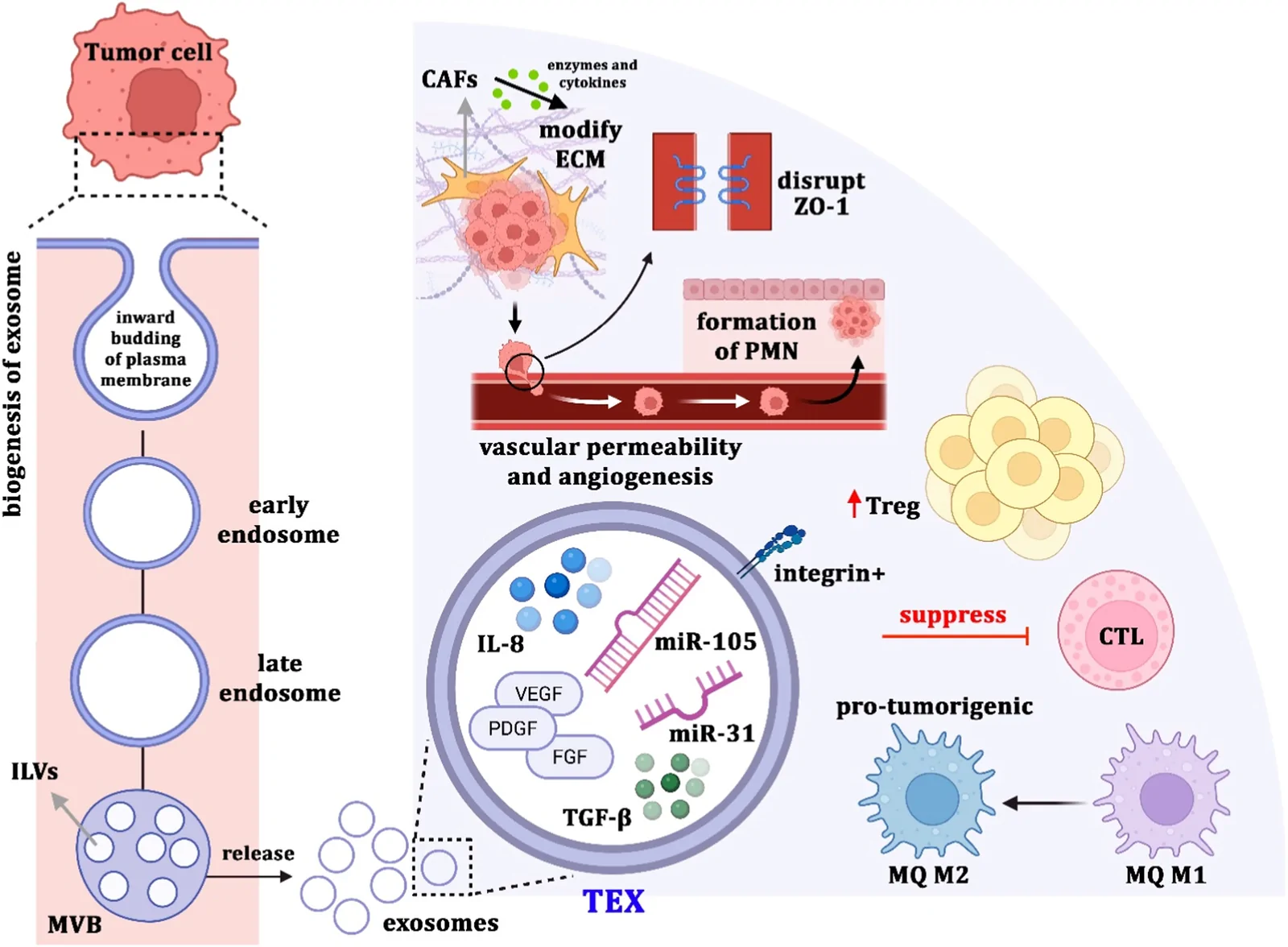
Biogenesis of tumor-derived exosomes (TEXs) and their role in breast cancer progression. The figure illustrates the biogenesis of exosomes, beginning with inward budding of the plasma membrane to form early endosomes, followed by their maturation into late endosomes and multivesicular bodies (MVBs) containing intraluminal vesicles (ILVs). Fusion of MVBs with the plasma membrane subsequently releases ILVs as exosomes. Tumor-derived exosomes (TEXs) carry diverse bioactive molecules, including cytokines such as IL-8 and TGF-β, growth factors such as VEGF, PDGF, and FGF, and tumor-promoting microRNAs such as miR-105 and miR-31. These cargos contribute to extracellular matrix (ECM) remodeling through the activation of cancer-associated fibroblasts (CAFs), disruption of endothelial tight junctions, increased vascular permeability, and angiogenesis. In particular, exosomal miR-105 targets zonula occludens-1 (ZO-1), thereby disrupting endothelial barrier integrity and facilitating tumor-cell dissemination. TEX-derived TGF-β contributes to immune suppression by promoting regulatory T cell (Treg) expansion and suppressing cytotoxic T lymphocyte (CTL) activity, while TEX-mediated signaling can promote macrophage polarization from the antitumor M1 phenotype toward the protumorigenic M2 phenotype. Collectively, these mechanisms facilitate pre-metastatic niche (PMN) formation, immune evasion, tumor growth, and metastatic progression in breast cancer. This figure was created by the authors using BioRender.com.

TEXs also actively regulate stromal components of the breast cancer microenvironment. These vesicles can induce fibroblast activation and promote the acquisition of cancer-associated fibroblast (CAF)-like properties, leading to extracellular matrix remodeling and enhanced tumor invasion. Furthermore, TEX-derived angiogenic factors, including vascular endothelial growth factor (VEGF), fibroblast growth factor (FGF), platelet-derived growth factor (PDGF), and IL-8, contribute to vascular formation and tumor expansion [45,46].

A major contribution of TEXs to breast cancer progression is immune suppression. TEXs transport immunoregulatory molecules such as (TGF-β, immune checkpoint-related proteins, and regulatory RNAs that impair effective antitumor immunity. These vesicles can suppress cytotoxic T lymphocyte activity, promote regulatory T cell expansion, and induce macrophage polarization toward a tumor-supportive M2 phenotype [47]. In addition, TEX-associated microRNAs, including miR-31, have been implicated in regulating invasive behavior and immune-related pathways that facilitate tumor progression.

The biological stability of TEXs is largely attributed to their lipid bilayer structure, which protects functional cargos from extracellular degradation and enables long-distance communication between tumor cells and distant tissues. Through this mechanism, TEXs can transfer oncogenic and immunomodulatory molecules, thereby contributing to breast cancer progression and resistance to immune-mediated elimination [48,49] (Fig. 1).

Although TEXs naturally promote tumor-supportive processes, their biological properties also provide important insights for therapeutic engineering. For example, understanding the mechanisms responsible for TEX-mediated tumor-cell recognition has inspired the development of engineered exosome systems designed to reproduce selective targeting while replacing immunosuppressive cargo with therapeutic molecules.

#### 2.3.2 Immune cell-derived exosomes as therapeutic platforms in breast cancer

In contrast to TEXs, EVs derived from immune cells can possess intrinsic immunostimulatory functions and have attracted considerable interest as natural platforms for cancer immunotherapy. Immune cell-derived EVs (IEXs) can transfer antigen-presenting molecules, immune regulatory proteins, and cytotoxic mediators, allowing modulation of both innate and adaptive immune responses.

Dendritic cell-derived EVs (DC-EVs) are among the most extensively investigated immune-derived vesicle platforms. These vesicles naturally express major histocompatibility complex (MHC) class I and class II molecules, together with co-stimulatory proteins such as CD86 and CD54, enabling antigen presentation and activation of tumor-specific T cell responses [40,50]. In breast cancer, DC-derived EVs have been explored as vaccine-like platforms capable of enhancing CD4^+^ and CD8^+^ T cell activation and promoting IFN-γ-mediated antitumor immunity.

Similarly, natural killer cell-derived EVs (NK-EVs) exhibit intrinsic cytotoxic activity due to the presence of effector molecules such as perforin, granzyme, and granulysin. These cargos enable NK-EVs to induce apoptosis in tumor cells while maintaining relatively low immunogenicity, supporting their potential application as therapeutic agents in aggressive breast cancer subtypes [40,50].

Recent studies have further demonstrated that EV-associated immune checkpoint molecules can be therapeutically exploited. For example, programmed cell death protein 1 (PD-1)-containing EVs released from activated immune cells can interact with PD-L1-expressing tumor cells, potentially interfering with PD-1/PD-L1-mediated immune suppression and restoring T cell activity [51]. These findings provide a conceptual basis for engineering EV surfaces with immune checkpoint-modulating molecules to enhance antitumor responses.

Beyond their direct immunological effects, immune-derived EVs can also serve as carriers for therapeutic modifications. For example, macrophage-derived exosomes have been investigated as components of targeted delivery systems in triple-negative breast cancer by exploiting macrophage membrane properties and tumor-associated receptor interactions [52]. Such approaches demonstrate how naturally occurring EV characteristics can be combined with engineering strategies to improve tumor targeting and therapeutic precision.

Despite their therapeutic promise, several challenges remain, including heterogeneity of EV populations, variability in cargo composition, potential off-target effects, and limitations in large-scale manufacturing. Comprehensive characterization, standardized production methods, and integration with other immunotherapeutic approaches, such as immune checkpoint blockade, will be necessary to maximize the clinical potential of immune-derived EV platforms [53].

#### 2.3.3 Biological rationale for engineered exosome-based breast cancer immunotherapy

The dual functions of tumor-derived and immune cell-derived EVs highlight the complexity of EV biology in breast cancer. TEXs promote tumor progression by enhancing metastatic niche formation, immune suppression, and microenvironmental remodeling, whereas IEXs provide endogenous mechanisms for antigen presentation, immune activation, and tumor cell killing.

This functional duality provides the rationale for engineered exosome-based immunotherapy: therapeutic platforms can be designed to retain favorable EV properties, including biocompatibility, biological targeting, and efficient cargo delivery, while introducing immune-stimulatory molecules, therapeutic RNAs, or anticancer agents. Therefore, breast cancer-specific understanding of EV biology represents a critical foundation for the engineering strategies and therapeutic applications discussed in the following sections.

## 3 Engineering strategies for exosomes

EV engineering involves the deliberate modification of EVs to enhance their functional properties, particularly for therapeutic applications. Various approaches are employed, including biological methods such as genetic manipulation of EV-producing cells to yield vesicles with desired traits; chemical techniques that enable covalent attachment of functional molecules to the EV surface to improve targeting; and physical methods like sonication, electroporation, extrusion, freeze–thaw cycles, membrane permeabilization, and hypotonic dialysis, primarily used to efficiently load therapeutic cargo [54].

### 3.1 Techniques for surface modification (e.g., targeting ligands, antibodies, aptamers)

Surface modification is critical to improving EV performance in biomedical contexts. Altering the surface enhances vesicle stability and reduces rapid clearance by the immune system. The use of biocompatible materials, such as polymers, minimizes nonspecific interactions and prolongs circulation time. Furthermore, conjugation of specific ligands, antibodies, peptides, or aptamers to the EV surface facilitates active targeting of specific cells or tissues, thereby increasing therapeutic efficacy while reducing systemic toxicity [55]. For example, engineered exosomes functionalized with anti-CD47 or anti-SIRPα antibodies have demonstrated potent tumoricidal effects in both in vitro and in vivo models [20,56]. Targeting ligands may include peptides like RGD, which bind integrins, or folic acid, which targets folate receptor-overexpressing cancer cells; these ligands enhance cellular uptake and limit off-target effects [57]. Aptamers, short single-stranded DNA or RNA oligonucleotides selected via SELEX (Systematic Evolution of Ligands by Exponential Enrichment), offer high specificity, low immunogenicity, and facile chemical modification, making them promising tools for targeted delivery and diagnostics [58]. Aptamer functionalization modifies EV uptake through clathrin-mediated endocytosis, improving cellular internalization [59]. Collectively, EV surface engineering strategies enhance nanoparticle stability, targeting specificity, and therapeutic precision (Fig. 2).

**Fig. 2:**
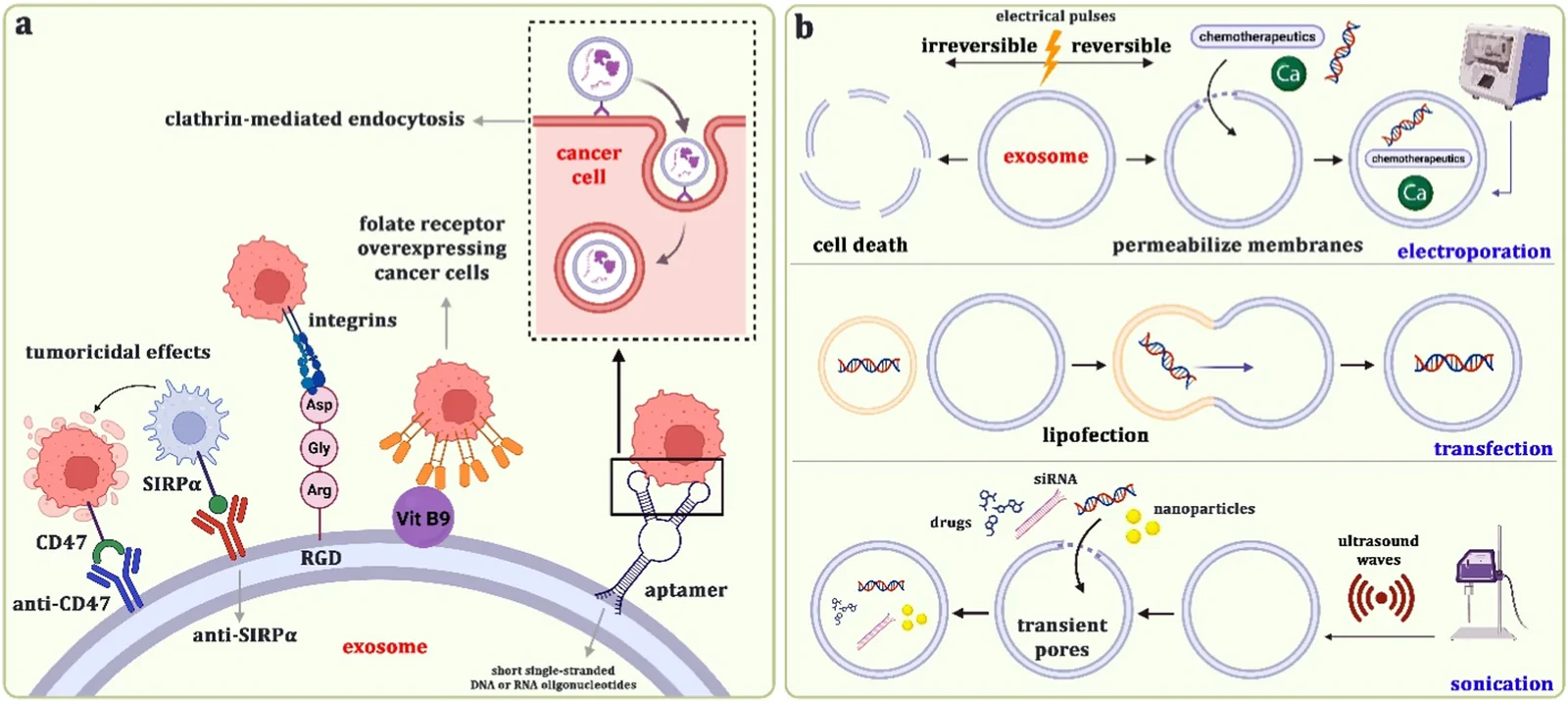
Strategies for engineering exosomes to enhance targeted delivery and therapeutic efficacy in cancer therapy. (a) Schematic illustration of surface modification strategies designed to improve exosome targeting and tumoricidal activity. Exosomes can be functionalized with specific ligands, including integrins containing Asp-Gly-Arg (DGR) motifs, aptamers consisting of short single-stranded DNA or RNA oligonucleotides, vitamins such as folate (vitamin B9), and antibodies such as anti-CD47 and anti-SIRPα. These surface modifications facilitate selective recognition and receptor-mediated internalization, including clathrin-mediated endocytosis, by cancer cells, thereby enhancing tumor-specific uptake, immune recognition, and cytotoxic activity. (b) Exogenous cargo-loading approaches, including electroporation, which uses electrical pulses to transiently permeabilize the exosomal membrane; lipofection, which facilitates the incorporation of nucleic acids through lipid-mediated delivery; and sonication, which uses ultrasound-induced transient membrane disruption to facilitate the loading of chemotherapeutic agents, siRNAs, or nanoparticles. Collectively, these engineering strategies enhance exosome targeting, cargo incorporation, cellular delivery, and therapeutic efficacy, supporting their application in precision oncology. This figure was created by the authors using BioRender.com.

### 3.2 Cargo loading methods (e.g., electroporation, transfection, sonication)

Cargo loading into EVs can be classified into cell-based and non-cell-based approaches. Cell-based loading involves introducing cargo molecules into donor cells, which are subsequently encapsulated naturally within EVs during biogenesis and secreted for therapeutic use [60]. Non-cell-based methods directly load isolated EVs using physical or chemical techniques such as electroporation, sonication, or transfection (referring here to direct transfection of isolated EVs rather than donor cells) [61]. Electroporation employs brief electrical pulses to transiently permeabilize membranes, enabling nucleic acid entry into the vesicles [62,63]. This method can be reversible, allowing temporary membrane permeabilization for delivery of chemotherapeutics, calcium, or DNA, or irreversible, causing permanent membrane damage and loss of vesicle integrity [64]. Transfection, in this non-cell-based context, uses chemical reagents (e.g., lipofection agents) to directly introduce nucleic acids or other cargo into purified EVs without involving donor cells. This approach is distinct from classical cell-based transfection, where foreign nucleic acids are introduced into eukaryotic cells to modify gene expression [55,65]. Sonication uses ultrasound waves to generate transient pores in EV membranes (sonoporation), facilitating cargo entry such as DNA, siRNA, drugs, or nanoparticles without chemical agents. Precise control of sonication parameters is essential to prevent vesicle disruption or structural damage [66,67] (Fig. 2).

### 3.3 Endogenous vs. exogenous engineering approaches

Cargo loading strategies are broadly divided into endogenous and exogenous methods. Endogenous loading involves genetic modification of donor cells to express and package therapeutic molecules such as proteins or RNAs into EVs during biogenesis, preserving membrane integrity and vesicle stability, though often yielding lower quantities with complex regulation [68]. Conversely, exogenous approaches load isolated EVs post-secretion via physical or chemical techniques (e.g., sonication, electroporation, freeze–thaw cycles, extrusion), achieving higher loading efficiency but potentially compromising vesicle structure or causing cargo aggregation [69].

The distinction between endogenous and exogenous engineering is particularly important because the timing of cargo incorporation can influence loading efficiency, vesicle integrity, cargo functionality, and reproducibility. Endogenous engineering enables therapeutic molecules to be incorporated during vesicle biogenesis and is therefore particularly suitable for genetically encoded cargos, including proteins, mRNAs, miRNAs, and other regulatory RNAs. Genetic modification of donor cells can also be used to alter the expression of membrane-associated proteins or targeting ligands, allowing cargo loading and surface functionalization to occur simultaneously [70]. Recent advances have further demonstrated that rational manipulation of protein–lipid interactions can enhance endogenous EV loading. Peruzzi et al. showed that proteins engineered to associate with plasma-membrane lipid rafts were preferentially incorporated into EVs and that this strategy could improve the functional delivery of engineered protein cargo to recipient cells. These findings provide a mechanistic framework for improving endogenous cargo sorting rather than relying solely on overexpression of therapeutic molecules [70].

Endogenous engineering has also become increasingly relevant to the delivery of genetically encoded therapeutic cargos and gene-editing systems. Donor cells can be modified to express therapeutic RNAs, proteins, or genome-editing components, which are subsequently incorporated into secreted EVs. Recent reviews have highlighted the potential of engineered EVs to transport CRISPR-associated components, while emphasizing that efficient packaging, tissue specificity, editing efficiency, and manufacturing reproducibility remain major challenges [71,72]. Importantly, recent experimental work has demonstrated that EV-mediated delivery of Cas9/sgRNA ribonucleoprotein complexes can achieve functional genome editing in cancer cells. Ye et al. developed genetically modified EVs with enhanced Cas9/sgRNA RNP loading and demonstrated target-gene editing and inhibition of proliferation in castration-resistant prostate cancer models, providing proof-of-concept for programmable gene editing using engineered EVs, although this approach has not yet been specifically validated as a breast cancer therapy [73].

In breast cancer, endogenous engineering has recently been applied directly to immunotherapeutic delivery. Aare et al. developed genetically engineered exosomes displaying anti-PD-L1 molecules on their surface and subsequently loaded them with STAT3 siRNA. In doxorubicin-resistant MDA-MB-231 models, these PD-L1-targeted exosomes enhanced antitumor activity when combined with paclitaxel, reduced tumor burden in vivo, and showed time-dependent tumor accumulation in both three-dimensional spheroids and xenograft models. The study also reported good tolerability in MCF-10A cells, illustrating how genetic surface engineering can be combined with exogenous nucleic-acid loading to generate multifunctional therapeutic vesicles for resistant triple-negative breast cancer [74].

Exogenous engineering, in contrast, is performed after EV isolation and provides greater flexibility for incorporating preformed therapeutic cargos. Passive incubation can be used for selected small molecules, whereas electroporation, sonication, extrusion, and freeze–thaw cycling facilitate the incorporation of larger or hydrophilic cargos, particularly nucleic acids [69,70]. However, these approaches may alter vesicle morphology, membrane integrity, aggregation, or cargo activity. For example, electroporation can facilitate nucleic-acid loading but may also promote cargo aggregation under inappropriate experimental conditions. Consequently, loading efficiency should not be considered the sole criterion for selecting an exogenous loading strategy; preservation of vesicle integrity and biological function must also be evaluated [69,70].

Recent breast cancer studies illustrate the therapeutic potential of post-isolation engineering. In a TNBC model, engineered EVs carrying miR-588 were generated by electroporation followed by cRGD surface modification using a post-insertion strategy. The resulting cRGD-EVs/miR-588 demonstrated enhanced TNBC targeting in vitro and in vivo and reduced immunosuppressive features of the tumor microenvironment through modulation of the CCL5-associated pathway [75]. These findings illustrate an important advantage of exogenous engineering: therapeutic RNA loading and surface targeting can be optimized sequentially after vesicle isolation, allowing greater modularity in the design of the final formulation.

Recent work has also expanded exogenous engineering beyond nucleic-acid loading toward combined drug loading and surface modification. In a 2025 breast cancer study, PH20-modified EVs loaded with curcumin were designed to improve penetration through the hyaluronan-rich tumor matrix. The engineered vesicles showed enhanced accumulation within the tumor core, increased CD8^+^ T cell infiltration, and improved antitumor activity in TNBC models, illustrating how post-isolation functionalization can be used to address physical and immunological barriers within the breast cancer microenvironment [76].

The modularity of exogenous engineering is also relevant when multiple therapeutic components are required. Surface ligands, targeting peptides, small-molecule drugs, and RNA cargos can be introduced sequentially or through combined procedures. Recent breast cancer-focused analyses have emphasized that the optimal loading strategy depends on the physicochemical properties of the cargo, the desired intracellular destination, and the intended therapeutic mechanism rather than on a universal superiority of endogenous or exogenous loading [77].

Overall, endogenous and exogenous approaches should be regarded as complementary engineering strategies rather than universally superior alternatives. Endogenous engineering provides greater opportunities for programmable control of cargo expression, intracellular sorting, and membrane composition, whereas exogenous engineering offers flexibility for loading diverse therapeutic molecules into preformed EVs and permits post-isolation surface modification. Recent breast cancer studies demonstrate that these strategies can be combined to generate multifunctional EV-based formulations capable of targeted delivery, immune modulation, and tumor-microenvironment remodeling. Nevertheless, differences in donor-cell source, cargo composition, loading efficiency, vesicle heterogeneity, and manufacturing conditions remain important barriers to reproducible clinical translation [68,69,77].

### 3.4 Hybrid systems (e.g., exosome-liposome hybrids, synthetic vesicles)

EVs, characterized by their lipid bilayer membrane, have emerged as promising nanocarriers for drug delivery [78]. Nonetheless, clinical translation of native EVs is hindered by challenges including scalability, lack of standardized isolation methods, limited cargo loading capacity, stability issues, and batch variability [79]. To address these limitations, artificial EV-based systems have been developed, encompassing nanovesicles (NVs), EV mimetics, and hybrid EVs, produced via top-down, bottom-up, and biohybrid engineering strategies respectively [69,80].

Hybridization of EVs with liposomes has attracted particular interest because it combines complementary properties of biologically derived and synthetic vesicles. Liposomes offer relatively high and controllable cargo-loading capacity, facile surface modification, compositional tunability, and established manufacturing technologies, whereas EV membranes provide biocompatibility, reduced immunogenicity, membrane-associated targeting properties, and efficient cellular interactions [81]. Fusion can be achieved using physical approaches such as sonication or freeze–thaw cycling, allowing integration of EV and liposomal membranes while retaining essential membrane characteristics. Consequently, hybridization may improve cargo incorporation, physicochemical stability, cellular uptake, controlled release, and in vivo persistence compared with some native EV formulations [[80], [81], [82], [83]].

Importantly, preclinical studies have provided direct evidence for the therapeutic potential of EV–liposome hybrids in breast cancer. In a 4T1 murine breast cancer model, tumor-derived EVs were fused with long-circulating, pH-sensitive liposomes containing doxorubicin (ExoSpHL-DOX). The hybrid formulation exhibited high drug encapsulation and pH-dependent drug release and showed antitumor activity in vivo. Notably, ExoSpHL-DOX produced lower systemic toxicity than free doxorubicin and reduced pulmonary metastatic foci, supporting the potential of EV–liposome fusion to improve the therapeutic index of conventional chemotherapy. These findings suggest that incorporation of liposomal characteristics into EV-based carriers can enhance drug delivery while retaining biologically relevant properties of the EV membrane [84].

More recent work has further extended this concept toward breast cancer theranostics. A 2025 study developed EV-fused liposomes containing therapeutic and imaging components and demonstrated improved physicochemical stability, cargo loading, sustained release, cellular uptake, and intracellular delivery compared with conventional formulations. In breast cancer models, the hybrid vesicles showed enhanced tumor accumulation and therapeutic activity, including increased apoptosis and suppression of tumor growth, while simultaneously enabling imaging-based assessment of tumor localization. These findings highlight an important advantage of hybridization: the synthetic liposomal component can provide greater control over cargo loading and release, whereas the EV membrane can contribute biological compatibility and tumor-interacting properties [85].

The potential of EV–liposome hybrids is particularly relevant to triple-negative breast cancer, where limited targetable receptors and an immunosuppressive tumor microenvironment frequently restrict therapeutic efficacy. In a recent TNBC study, EV–liposome hybrid nanoparticles were engineered to co-deliver a photothermal agent and a STING agonist. The EV membrane component incorporated characteristic membrane proteins, including CD9 and CD63, while the liposomal hybrid platform enabled incorporation of the therapeutic cargo. Upon near-infrared irradiation, the system induced photothermal effects and stimulated controlled release of the STING agonist, promoting type I interferon signaling and antitumor immune activation. Importantly, treatment suppressed both primary and distant tumor growth and promoted immune memory, illustrating how hybridization can integrate targeted delivery, controlled drug release, photothermal therapy, and immune stimulation within a single platform [86].

Beyond EV–liposome fusion, other biohybrid platforms have been investigated to improve therapeutic performance in breast cancer. EV membranes can be combined with synthetic nanoparticles or other engineered cores to exploit the membrane-mediated biological interactions of exosomes while benefiting from the greater structural stability and cargo capacity of synthetic nanomaterials. Such systems can facilitate controlled delivery of chemotherapeutics, nucleic acids, photosensitizers, or immunomodulatory agents and may provide greater flexibility in therapeutic design than native EV alone [87].

From a translational perspective, hybrid systems may therefore offer several advantages over native EVs. First, the synthetic component can increase and standardize cargo loading, addressing one of the major limitations of naturally occurring EVs. Second, liposomal and synthetic components can be chemically modified to introduce stimulus-responsive release, targeting ligands, or imaging functions. Third, hybridization may improve formulation stability and reproducibility and potentially facilitate scalable manufacturing. In contrast, native EVs exhibit substantial heterogeneity in size, membrane composition, cargo content, and biological activity, and their production and purification remain difficult to standardize [88,89]. Thus, hybrid platforms may provide a practical compromise between the biological functionality of EV membranes and the engineering flexibility of synthetic nanocarriers.

Nevertheless, these advantages should not be interpreted as evidence that hybrid systems universally outperform native EVs. The biological activity of EV membranes depends strongly on their cellular origin, and fusion processes may alter membrane proteins, surface topology, biodistribution, or biological interactions. Moreover, differences in preparation methods can substantially influence the physicochemical and functional properties of hybrid vesicles. Standardized characterization, comparative pharmacokinetic studies, long-term safety assessment, and head-to-head evaluation against native EVs and conventional liposomes are therefore required before their clinical potential can be established [90].

Overall, current preclinical evidence supports EV–liposome hybridization as a promising strategy for overcoming several limitations of native EVs while preserving selected biological advantages of exosomal membranes. The ability to combine controlled cargo loading and release with biocompatibility, tumor interaction, multimodal functionality, and immune stimulation makes hybrid systems particularly attractive for breast cancer, including TNBC. Further optimization of manufacturing, membrane composition, targeting strategies, and therapeutic cargo selection will be essential for translating these multifunctional platforms from experimental models into clinically relevant EV-based therapies.

## 4 Applications in breast cancer immunotherapy

### 4.1 Delivery of immune checkpoint inhibitors (e.g., PD-L1 siRNA, anti-CTLA-4)

Among current cancer treatment strategies, immunotherapy, particularly immune checkpoint blockade (ICB) has emerged as a transformative approach. Checkpoint pathways regulate immune activation, and tumors often exploit them to suppress antitumor immune responses and evade immune detection [91]. Inhibitors such as anti-CTLA-4 antibodies and PD-L1-targeting siRNAs have shown promise in enhancing immune specificity, efficacy, and reducing adverse effects in breast cancer immunotherapy [92].

Targeted and sustained delivery of immune checkpoint inhibitors (ICIs) in breast cancer has been shown to enhance therapeutic efficacy while minimizing systemic toxicity. Local activation of immune responses can transform immunologically "cold" tumors into more immunogenic ones. Various delivery systems such as hydrogels, viral vectors, platelets, and nanoparticles, are being explored to optimize ICI distribution. Combination therapies and tailored delivery platforms are critical to achieving effective immunomodulation [93,94].

For example, dextran-based biodegradable nanoparticles carrying PD-L1 siRNA have been used in TNBC models to silence PD-L1 expression, thereby restoring immune recognition and enabling effective cytotoxic responses [95]. As siRNA-based therapeutics are highly sequence-specific, they significantly expand the scope of druggable targets, making them attractive tools in precision oncology [96].

Anti-CTLA-4 therapies aim to overcome immune suppression by activating T cells and depleting Tregs. In breast cancer, combination strategies involving targeted delivery of anti-CTLA-4 agents have improved efficacy while reducing immune-related adverse events [97]. CTLA-4 expression on certain breast cancer cells inhibits T cell and DC function. Its blockade restores DC activity, enhances antigen presentation, and promotes activation of CD4^+^ and CD8^+^ T cells. Moreover, CTLA-4 inhibition may exert direct antitumor effects by inducing apoptosis in CTLA-4-expressing tumor cells [98].

### 4.2 Exosome-based cancer vaccines (tumor antigens, neoantigens)

Small extracellular vesicles (small EVs), including vesicles commonly described as exosomes when an endosomal origin has been established, are nanoscale membrane-bound vesicles that carry proteins, lipids, and nucleic acids and participate in intercellular communication [99,100]. DEXs are particularly relevant to immune modulation because they can display antigen-presenting and co-stimulatory molecules and activate immune responses [101]. Engineered DEXs loaded with tumor-associated antigens or neoantigens can elicit robust cytotoxic T cell responses while minimizing off-target effects. Due to their natural biocompatibility and ability to traverse biological barriers such as the blood-brain barrier, these vesicles represent an effective platform for antigen delivery [102,103].

Patient-specific neoantigen-loaded DEXs have demonstrated the ability to induce both T and B cell responses in melanoma and colorectal cancer models, suggesting their potential utility in breast cancer as well [103,104]. These vaccines leverage the biological properties of dendritic cell-derived vesicles to enhance immunogenicity, reprogram the tumor microenvironment, and potentially improve treatment outcomes in breast cancer [102,105].

Exosome-derived immunomodulatory signals can also contribute to the reprogramming of immunosuppressive elements in the tumor milieu. For instance, OX40L-expressing EVs can convert tumor-associated macrophages (TAMs) from an M2 (pro-tumor) to an M1 (anti-tumor) phenotype, thereby enhancing inflammatory signaling and phagocytic activity [105]. Additionally, in TNBC, engineered vesicles targeting the lncRNA SNHG16/miR-16-5p axis have been shown to inhibit γδ T cell proliferation and reduce secretion of immunosuppressive cytokines such as IL-17 and TGF-β, further strengthening antitumor immunity [102].

### 4.3 Modulation of tumor-associated macrophages (TAMs) and dendritic cells

TAMs are a dominant immune cell population in solid tumors and play a critical role in shaping the immunosuppressive microenvironment [106]. Owing to their plasticity, macrophages can shift from an anti-tumoral M1 phenotype to a tumor-promoting M2 phenotype under tumor-derived signals. M2 macrophages facilitate immune evasion, angiogenesis, and metastasis, thus contributing to resistance against immunotherapy [107].

Therapeutic strategies targeting TAMs typically focus on four main approaches: depletion, reprogramming, inhibition of monocyte recruitment, and blockade of suppressive signaling molecules. Reprogramming TAMs toward an M1 phenotype is particularly effective in enhancing immune responses against breast cancer [106,108].

The tumor microenvironment also impairs dendritic cell function, leading to poor antigen presentation and ineffective T cell priming in breast cancer [109]. Dendritic cell-based vaccines are emerging as a promising immunotherapeutic strategy, as they elicit robust tumor-specific T cell responses and have demonstrated encouraging clinical efficacy [110].

Targeted delivery of tumor antigens to dendritic cells via surface receptors such as DEC205 can potentiate immune responses. For instance, targeting HER2 to dendritic cells enhances antigen presentation and induces sustained CD4^+^ and CD8^+^ T cell responses. Incorporating adjuvants like TLR agonists further supports DC maturation and immune activation [111,112].

To overcome tumor-induced DC dysfunction, strategies involving inhibition of immunosuppressive signaling pathways and TLR activation are being investigated. When combined with checkpoint inhibitors or cancer vaccines, these methods can significantly enhance the immune response in breast cancer [113].

### 4.4 Inducing T cell and NK cell activation using engineered exosomes

Extracellular vesicles released through the endosomal pathway contain diverse biomolecules essential for immune signaling and intercellular communication [98]. The engineering of these vesicles offers a novel avenue to enhance their therapeutic utility by enabling the incorporation of specific ligands, drugs, or genetic material [114]. For example, engineered EVs expressing both anti-CD3 (targeting T cells) and anti-HER2 (targeting breast cancer cells) antibodies have shown the capacity to bridge tumor and immune cells, facilitating cytotoxic T lymphocyte (CTL) activation and effective tumor targeting [115].

Engineered vesicles expressing high-affinity PD-1 variants can block PD-L1-mediated T cell suppression, restoring T cell function in the tumor microenvironment [116]. Additionally, EVs enriched with immunomodulators such as OX40L and CD62L have demonstrated potential to enhance effector T cell activity and reduce Treg-mediated suppression [102]. NK cell-derived EVs carry cytotoxic proteins such as perforin and granzyme, as well as surface markers enabling tumor targeting. These vesicles exhibit selective cytotoxicity against breast cancer cells while sparing normal tissues [117].

Furthermore, NK cell-derived EVs can be loaded with chemotherapeutic agents such as cisplatin to enhance their antitumor efficacy and overcome drug resistance. FasL-expressing NK-derived EVs promote apoptosis in Fas-positive tumor cells and modulate the immune response by cytokine release, thereby enhancing both innate and adaptive immunity [118,119] (Fig. 3).

**Fig. 3:**
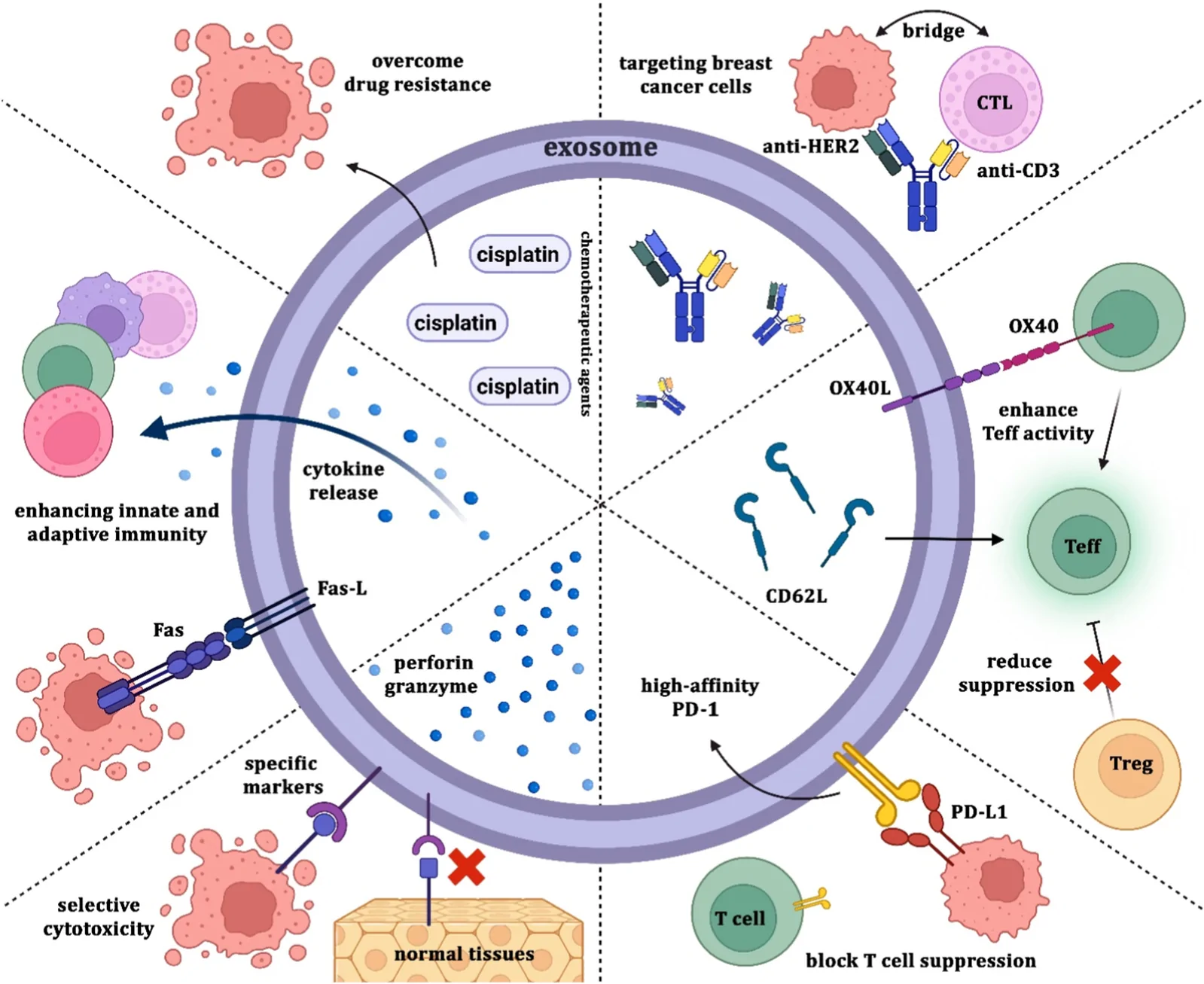
Mechanistic roles of engineered exosomes in breast cancer immunotherapy. Engineered exosomes exhibit multifaceted immunotherapeutic functions, including: (i) targeted delivery of therapeutic agents, such as cisplatin, to enhance antitumor activity and overcome chemoresistance; (ii) tumor-specific targeting through ligands such as anti-HER2 antibodies, which facilitate interactions between cytotoxic T lymphocytes (CTLs) and HER2-expressing breast cancer cells; (iii) T cell activation and reversal of immunosuppression through OX40L-mediated enhancement of effector T cell (Teff) activity and high-affinity PD-1 variants that interfere with PD-L1-mediated immune suppression; (iv) selective tumor-cell cytotoxicity through FasL–Fas interactions and the release of cytotoxic molecules, including perforin and granzymes; (v) enhancement of innate and adaptive antitumor immunity through cytokine-mediated signaling and CD62L-associated immunomodulation; and (vi) reduction of off-target toxicity through preferential targeting and sparing of normal tissues. Collectively, these engineered vesicles integrate targeted drug delivery, immune activation, and immune checkpoint modulation to enhance the therapeutic potential of breast cancer immunotherapy. This figure was created by the authors using BioRender.com.

Engineered EVs and exosome-derived platforms, due to their potentially low immunogenicity and inherent biological interactions with recipient cells, represent promising modalities for activating T cells and NK cells while reshaping the immunosuppressive tumor microenvironment. Their multifunctional potential makes them attractive candidates for next-generation immunotherapies in breast cancer [114,117].

Collectively, the therapeutic applications described above highlight the multifunctional potential of engineered extracellular vesicles, including engineered exosome-based platforms, in breast cancer immunotherapy. Rather than functioning as isolated approaches, these vesicle-based strategies may complement one another by simultaneously enhancing antigen presentation, modulating immunosuppressive immune cells, promoting T- and NK-cell activation, and overcoming immune checkpoint-mediated suppression. In particular, combining vesicle-based cancer vaccines with immune checkpoint blockade may provide complementary mechanisms to enhance tumor antigen recognition and sustain antitumor immune responses. Similarly, integration of EV-mediated immune cell modulation with targeted delivery of immunomodulatory cargos may further improve the efficacy of immunotherapy by reshaping the tumor microenvironment. These combinatorial strategies provide a rationale for developing multifunctional EV-based platforms and represent an important direction for future translational studies in breast cancer immunotherapy.

## 5 Advantages of engineered exosomes over traditional nanocarriers

Exosomes, as a defined subset of EVs, are naturally occurring membrane-bound vesicles surrounded by a lipid bilayer and enriched with membrane proteins, lipids, and diverse biological cargos. These characteristics provide several potential advantages for therapeutic delivery, including efficient interactions with recipient cells, protection of encapsulated molecules from extracellular degradation, and the possibility of exploiting endogenous membrane components for cellular targeting [120,121]. Unlike conventional synthetic nanoparticles, biologically derived EVs possess surfaces that can participate in receptor-mediated interactions, membrane fusion, and intracellular trafficking. Nevertheless, these properties should not be interpreted as evidence that exosome-based or EV-based systems are universally superior to synthetic nanocarriers. Their therapeutic performance is highly dependent on the cellular source, cargo, engineering strategy, administration route, and physicochemical characteristics of the vesicles [122,123].

An important consideration when comparing native EVs with synthetic nanocarriers is their pharmacokinetic behavior. Although their endogenous origin is frequently associated with prolonged circulation and reduced immunogenicity, experimental evidence indicates that unmodified EVs can undergo rapid systemic clearance. Charoenviriyakul et al. compared EVs described as exosomes derived from five different murine cell lines and demonstrated that all EV preparations rapidly disappeared from the systemic circulation after intravenous administration, with predominant accumulation in the liver [124]. These findings challenge the assumption that biological origin alone inherently confers prolonged circulation and indicate that EV biodistribution is strongly influenced by biological and physicochemical characteristics. Thus, endogenous origin alone does not guarantee reduced clearance or improved systemic tumor accumulation.

Direct comparisons with synthetic liposomes further support a context-dependent interpretation of EV-based advantages. Smyth et al. compared unmodified tumor-derived EVs with conventional phosphatidylcholine/cholesterol (PC:Chol) liposomes and liposomes prepared from EV-derived lipid extracts [125]. Following intravenous administration, all three formulations exhibited rapid clearance from the circulation and relatively limited tumor accumulation. These findings indicate that the endogenous protein and lipid composition of tumor-derived EVs does not necessarily confer superior systemic pharmacokinetics or tumor targeting compared with conventional liposomal formulations. Interestingly, following intratumoral administration, tumor-derived EVs showed significantly greater retention within tumor tissue than PC:Chol liposomes [125]. Therefore, the potential advantage of EV-based systems may depend substantially on the administration route and biological context rather than representing an intrinsic superiority over synthetic liposomes.

Surface engineering provides an opportunity to overcome some of these limitations by actively controlling EV biodistribution. Hwang et al. demonstrated that chemical modification of EV surface properties substantially altered their in vivo distribution and clearance patterns [126]. Zwitterionic fluorophore-labeled EVs exhibited rapid renal clearance with relatively low nonspecific tissue accumulation, whereas anionic EVs showed predominant hepatobiliary elimination and pronounced liver accumulation. Importantly, the pharmacokinetic and biodistribution profiles of the modified EV conjugates were comparable to those of the corresponding free fluorophores [126]. These findings emphasize that surface chemistry is an important determinant of EV fate in vivo and support rational engineering as a strategy to tailor biodistribution rather than relying exclusively on the intrinsic properties of native EVs.

Another potential advantage of engineered EVs, including engineered exosome-based systems, is their capacity to transport diverse classes of biological cargos while facilitating interactions with recipient cells. Naturally occurring EVs can contain proteins and nucleic acids, including mRNAs, miRNAs, and siRNAs, whereas engineering approaches can further increase cargo loading or introduce therapeutic molecules and targeting ligands [127,128]. The vesicular membrane can protect labile cargos from extracellular degradation and may facilitate intracellular delivery following uptake. However, synthetic nanoparticles retain important advantages, including precisely controlled composition, reproducible physicochemical properties, established manufacturing procedures, high loading capacity for selected compounds, and greater potential for large-scale production. Consequently, comparisons between the two platforms should consider the intended therapeutic application rather than assuming universal superiority of one system over the other. Representative quantitative and preclinical evidence comparing EV-based systems, including exosome-derived formulations, with conventional nanocarriers is summarized in Table 1.

**Table 1:** Quantitative and preclinical evidence supporting the therapeutic advantages of engineered exosomes.

Study	Model/strategy	Quantitative or experimentally defined outcome	Relevance to engineered exosomes
Charoenviriyakul et al. [124]	Native exosomes from multiple murine cell lines; systemic administration	Rapid systemic clearance with circulation half-life of approximately 2–4 min and predominant hepatic distribution	Demonstrates that native exosomes are not intrinsically long-circulating
Smyth et al. [125]	Tumor-derived exosomes vs PC:Chol liposomes and exosomal-lipid liposomes	Comparable rapid systemic clearance and limited tumor accumulation after IV administration; greater tumor retention of exosomes after intratumoral administration	Shows that exosomes are not universally superior to liposomes and that administration route is important
Hwang et al. [126]	Chemically engineered exosomes with different surface properties	Surface modification substantially altered biodistribution and clearance pathways, with zwitterionic and anionic formulations showing distinct renal vs hepatobiliary elimination	Demonstrates that engineering can actively control exosome biodistribution
Qiao et al. [129]	Tumor-derived exosomes and Doxil-loaded exosomes	Tumor-derived exosomes preferentially fused with parental cancer cells and homed to the original tumor; Doxil-loaded exosomes showed enhanced tumor retention and therapeutic efficacy compared with Doxil alone	Supports biological tumor homing and improved local drug delivery
O'Brien et al. [130]	MSC-engineered EVs enriched with miR-379; breast cancer models	Systemically administered cell-free miR-379-enriched EVs produced a therapeutic effect; reduced tumor formation/growth was observed in miR-379 breast cancer models	Provides direct breast cancer evidence for engineered EV-mediated RNA delivery
Paul et al. [131]	Doxorubicin-loaded dendritic-cell-derived exosomes (ExoDS); MDA-MB-231	IC_50_: 74.28 ng for ExoDS vs 164.11 ng for liposomal doxorubicin and 145.62 ng for free doxorubicin	Direct quantitative evidence of enhanced drug potency in breast cancer
Yang et al. [132]	Cellular nanoporation for production of mRNA-loaded exosomes	Up to 50-fold greater exosome production and >10^3^-fold increase in exosomal mRNA transcripts compared with conventional approaches	Demonstrates an engineering strategy addressing cargo loading and manufacturing limitations
Romagnoli et al. [133]	DC-derived exosomes; SK-BR-3 breast cancer cells	DC-exosome-treated tumor cells induced significantly greater IFN-γ-producing T cell responses than untreated tumor cells	Supports immunomodulatory and vaccine-related potential of exosomes

Importantly, quantitative evidence from breast cancer models provides a more direct basis for evaluating the therapeutic advantages of engineered EVs. Paul et al. developed doxorubicin-loaded vesicles derived from mature dendritic cells (ExoDS) and compared their cytotoxic activity with free doxorubicin and liposomal doxorubicin in breast cancer models [131]. In MDA-MB-231 cells, ExoDS exhibited an IC_50_ of 74.28 ng, compared with 164.11 ng for liposomal doxorubicin and 145.62 ng for free doxorubicin, indicating substantially greater in vitro cytotoxic potency under the experimental conditions of the study [131]. Moreover, ExoDS at 5 ng/mL produced approximately comparable apoptotic effects to free doxorubicin at 1.8 μg/mL in breast cancer cells and cancer stem cells derived from MCF-7, MDA-MB-468, and MDA-MB-231 models. This corresponds to an approximately 400-fold lower concentration of the vesicle-associated formulation required to achieve a comparable apoptotic response under the reported experimental conditions [131]. Importantly, ExoDS preferentially affected breast cancer cells and cancer stem cells, whereas no significant apoptosis was detected in healthy mammary cells or peripheral blood mononuclear cells in the same study [131]. These findings provide quantitative preclinical evidence that an EV-based formulation can enhance cytotoxic activity and cellular selectivity compared with free and liposomal doxorubicin.

The same study also provides evidence that the biological origin and surface composition of EVs can influence cellular uptake. Fluorescence-based analyses demonstrated significantly greater internalization of ExoDS by MDA-MB-231 breast cancer cells and their derived cancer stem cells at 6 h, whereas control EVs derived from THP1 cells showed a later uptake pattern, predominantly at approximately 12–24 h [131]. These observations suggest that vesicle engineering and donor-cell selection can influence the interaction of EVs with tumor cells and may provide an opportunity to improve intracellular delivery. However, these findings should be interpreted within the specific experimental context, as enhanced uptake in vitro does not necessarily translate into improved tumor accumulation or therapeutic efficacy following systemic administration.

The ability of tumor-derived EVs, including exosome-enriched preparations, to interact with tumor cells can also be exploited for selective drug delivery. Qiao et al. demonstrated that tumor cell-derived EVs described as exosomes preferentially home to their cells of origin and proposed their use as Trojan horse carriers for anticancer drugs [129]. Such homotypic interactions may provide a biologically relevant targeting mechanism that is difficult to reproduce using conventional nanoparticles without additional surface functionalization. Nevertheless, the extent to which this phenomenon can be translated into clinically meaningful tumor targeting remains dependent on the vesicle source, tumor type, administration route, and in vivo biodistribution profile.

Beyond small-molecule drugs, engineered EV-based platforms can transport immunogenic proteins and nucleic acids, supporting their application in cancer immunotherapy. Dendritic cell-derived exosomes carrying tumor-associated peptides in association with MHC-I molecules can stimulate cytotoxic T lymphocyte and NK-cell responses, providing a mechanistic basis for their use as cancer vaccine platforms [133]. EV-mediated delivery has also been investigated for multiple classes of nucleic acids, including mRNAs, miRNAs, siRNAs, and other regulatory RNAs [134]. Large-scale generation of functional mRNA-encapsulating EVs with exosomal characteristics has further demonstrated the feasibility of engineering biologically derived vesicles for nucleic acid delivery, highlighting their potential as RNA delivery vehicles [132].

In breast cancer, the therapeutic potential of EV-mediated RNA delivery has also been demonstrated. O'Brien et al. reported that mesenchymal stem cell-supported EVs enriched with miR-379 could be exploited for tumor-targeted delivery and demonstrated therapeutic activity in breast cancer models [130]. Similarly, small EVs derived from human umbilical cord mesenchymal stromal cells and enriched with exogenous miR-145-5p inhibited pancreatic ductal adenocarcinoma progression in vitro and in vivo, illustrating the broader potential of EV-mediated miRNA delivery across cancer models [135]. In addition, Shtam et al. demonstrated that exosome preparations can serve as carriers of exogenous siRNA, including RAD51-targeting siRNA, supporting their potential utility for RNA interference-based therapeutic strategies [136]. Although these studies provide proof-of-concept evidence, differences in vesicle nomenclature, tumor type, vesicle source, loading strategy, and experimental model should be considered when extrapolating their findings to breast cancer immunotherapy.

Taken together, the available evidence indicates that the advantages of engineered exosome-based systems and EV-derived platforms over traditional nanocarriers are context-dependent rather than absolute. Native EV preparations do not necessarily exhibit prolonged circulation, reduced hepatic accumulation, or superior systemic tumor targeting compared with synthetic nanoparticles or liposomes [124,125]. Instead, their principal therapeutic value may arise from the combination of a biologically derived membrane, endogenous mechanisms of cellular interaction, protection of complex biological cargos, and the possibility of rational surface and cargo engineering. Quantitative evidence in breast cancer models, particularly the ExoDS study, demonstrates enhanced in vitro cytotoxic potency compared with both free and liposomal doxorubicin, with an IC_50_ of 74.28 ng versus 145.62 and 164.11 ng, respectively [131]. At the same time, pharmacokinetic studies emphasize that these advantages cannot be generalized to all administration routes or formulations [[124], [125], [126]]. Therefore, engineered exosomes should be viewed not as universally superior replacements for synthetic nanocarriers, but as complementary next-generation delivery platforms whose greatest potential may emerge when biological targeting, efficient intracellular delivery, immune modulation, and the simultaneous delivery of complex therapeutic cargos are required. Future head-to-head studies using standardized formulations, matched doses, and clinically relevant in vivo breast cancer models will be essential to determine when engineered exosomes provide a genuine therapeutic advantage over established nanocarrier technologies.

## 6 Preclinical and clinical developments

Compared to chimeric antigen receptor T (CAR-T) cell therapy, the administration of CAR-T cell-derived EVs has demonstrated a favorable safety profile in preclinical models of cytokine release syndrome, suggesting that engineered EV-based platforms derived from CAR-T cells may offer a safer and potentially promising alternative for the treatment of breast cancer [114]. Yang et al. showed that anti-mesothelin (MSLN) CAR-T cell-derived EVs significantly inhibited tumor growth in vivo and selectively eradicated MSLN-positive triple-negative breast cancer cells without inducing systemic toxicity. These CAR-T-derived vesicles, similar to their parent cells, carried granzyme B and perforin, exhibiting dose-dependent anti-tumor effects, thus presenting a potential alternative approach to conventional CAR-T therapies [137].

In xenograft models of breast cancer, intravenous administration of HER2-targeted exosome preparations loaded with miR-34a (tEx) improved tumor targeting and exerted potent anti-tumor effects. This therapy significantly downregulated the anti-apoptotic protein Bcl-xL, increased the expression of pro-apoptotic markers, and suppressed PD-L1 expression, collectively promoting cancer cell apoptosis and tumor suppression [138].

Hu et al. developed engineered extracellular vesicle-like nanovesicles (eNVs-FAP) designed to reprogram the tumor microenvironment (TME) by delivering fibroblast activation protein-alpha (FAP), functioning as tumor vaccines. These nanovesicles induced cytotoxic T lymphocyte (CTL) responses and inhibited tumor growth in multiple cancer models, including colon, melanoma, lung, and breast cancer [139].

Additional studies have explored the immunogenic potential of EV-based vaccine platforms. For example, DC-primed exosome preparations (HELA-Exos) loaded with immunogenic cell death (ICD) inducers, including Hiltonol (a TLR3 agonist) and human neutrophil elastase (ELANE), enhanced immune responses in breast cancer models by activating cDC1s and promoting CD8^+^ T cell-mediated immunity [22]. Muzaffer et al. introduced two immunoadjuvants -a TLR9 ligand (K-type CpG ODN) and a TLR3 ligand (poly(I:C))- into breast cancer cell-derived exosomes. These engineered exosomes enhanced antigen-specific T cell responses and effectively suppressed tumor growth in vivo [140].

Wu et al. demonstrated that combining IL-2-modified DC-derived exosomes with immune checkpoint inhibitors (ICIs) sensitized breast tumors to immunotherapy. Mechanistically, this combination increased the proportions of activated T cells and tumor-infiltrating lymphocytes, providing a synergistic approach for managing metastatic breast cancer [141].

Wan et al. and Wang et al. successfully loaded paclitaxel (PTX) and let-7 microRNAs into DC-derived exosomes modified with polyethylene glycol or AS1411 aptamer-conjugated polypeptides, which target nucleolin overexpressed on breast cancer cells [142,143]. To enhance tumor specificity, another team engineered immature murine DC-derived exosomes with a Lamp2b-iRGD fusion protein and electroporated them with doxorubicin (DOX), achieving 20% drug encapsulation efficiency. These engineered vesicles specifically targeted αv integrin-positive breast cancer cells and exhibited a low-toxicity therapeutic profile [144]. Similarly, Gomari et al. utilized LAMP2b-DARPin-modified exosomes to deliver DOX specifically to HER2-positive breast cancer cells [145].

Peng et al. engineered red blood cell (RBC)-derived EVs to encapsulate RIG-I agonists, which successfully targeted breast cancer cells and induced apoptosis in both human and murine models by activating the RIG-I signaling pathway [146]. NK cell-derived exosomes (NK-Exos) also exhibit strong anti-tumor effects due to their innate cytotoxic protein content, such as perforin and granzyme B, which can eliminate aggressive breast cancer subtypes including TNBC [147]. In a canine mammary tumor model, Lee et al. reported that NK-Exos reduced tumor volume and expression of tumorigenic markers such as CD133, Bmi-1, VEGF, and MMP-3, highlighting their potential to inhibit tumor progression and cancer stem cell survival [148].

Kaban et al. showed that NK-Exos carrying BCL-2-targeting siRNAs increased apoptosis in estrogen receptor-positive (ER^+^) breast cancer cells, potentially overcoming endocrine therapy resistance [149]. Tao et al. developed a multifunctional nanoplatform, ExoCAR/T7@Micelle, incorporating CAR-NK-derived exosomes for the treatment of HER2-positive breast cancer brain metastases. These engineered exosomes could traverse the blood–brain barrier, target HER2^+^ tumor cells, and deliver reactive oxygen species-amplified photodynamic therapy. In vivo, the platform significantly prolonged survival without evident toxicity [150].

Jung et al. targeted tumor hypoxia using tumor-derived exosome preparations from MDA-MB-231 cells, which were engineered with superparamagnetic iron oxide nanoparticles (SPIOs) and loaded with the PARP inhibitor Olaparib. This platform enhanced apoptosis and inhibited tumor progression in vivo [151]. Another study introduced EXO-DEPT, an exosome-based delivery system carrying mRNA encoding HChrR6 for HER2-overexpressing tumors. In combination with the prodrug CNOB, EXO-DEPT suppressed tumor growth in HER2-positive xenografts, indicating the feasibility of vesicle-mediated mRNA delivery for cancer therapy [152].

MSC-derived EVs containing miR-379, a known tumor suppressor that targets cyclooxygenase-2 (COX-2), demonstrated strong therapeutic efficacy against breast cancer in vivo, providing a novel option for metastatic breast cancer treatment [130].

A promising strategy involves the use of folic acid-modified exosomes encapsulating PH20 hyaluronidase (FA-Exo-PH20) to target the tumor microenvironment. These vesicles degrade hyaluronan (HA), reduce interstitial pressure, and promote M1 macrophage polarization, improving drug delivery and inhibiting metastasis [153]. Similarly, a combination therapy integrating small EV-PH20 (sEV-PH20) with PD-L1 checkpoint inhibition improved CD103^+^ dendritic cell recruitment and T cell infiltration into the tumor microenvironment, thereby enhancing immunotherapeutic efficacy [154].

Finally, a novel class of genetically engineered retargeted EV platforms, termed Synthetic Multivalent Antibodies Retargeted Exosomes (SMART-Exos), has been developed. These bispecific antibody-decorated vesicles simultaneously target HER2 on tumor cells and CD3 on T cells, enabling direct T cell activation. In preclinical models, SMART-Exos significantly suppressed HER2-positive breast cancer growth, representing a cutting-edge platform for targeted cancer immunotherapy [115].

Preclinical findings and innovative therapeutic approaches underscore the transformative potential of engineered exosome-based and EV-based platforms in breast cancer immunotherapy. These strategies not only demonstrate efficacy in modulating the tumor microenvironment but also pave the way for clinical translation. Notably, several ongoing clinical trials are currently investigating the therapeutic application of Preclinical findings and innovative therapeutic approaches underscore the transformative potential of engineered exosome-based and EV-based platforms in breast cancer immunotherapy. These strategies demonstrate efficacy in modulating the tumor microenvironment and provide a foundation for future clinical translation. Importantly, the current clinical landscape remains distinct from the preclinical literature: most registered breast cancer studies involving exosomes or sEVs primarily investigate diagnostic, prognostic, predictive, molecular subtyping, or biomarker applications rather than engineered exosome-based immunotherapy. Therefore, these studies should be interpreted as evidence of growing clinical interest and translational feasibility of exosome- and sEV-related technologies rather than as clinical validation of engineered exosome therapeutics. Representative clinical studies are summarized in Table 2 to provide an overview of the current clinical landscape and translational status of exosome- and sEV-based research in breast cancer.

**Table 2:** Representative clinical studies evaluating exosome- and small extracellular vesicle (sEV)-based applications in breast cancer.

Clinical Trial	Application	Study Title	Phase	Condition	Study Type	Current Status
NCT05955521	Prognostic/predictive biomarker	Exosome as the Prognostic and Predictive Biomarker in EBC Patients	N/A	TNBC; HER2-positive breast cancer	Interventional	Active, not recruiting
NCT01344109	Diagnostic/prognostic biomarker	A Pilot Study of Tumor-Derived Exosomes as Diagnostic and Prognostic Markers in Breast Cancer Patients Receiving Neoadjuvant Chemotherapy	N/A	Breast neoplasms	Observational	Completed/terminated study record
NCT05286684	Diagnostic/monitoring	Feasibility of Exosome Analysis in Cerebrospinal Fluid During the Diagnostic Workup of Metastatic Meningitis From Breast Cancer	N/A	Breast cancer; suspected leptomeningeal metastasis	Interventional	Completed
NCT03974204	Diagnostic/monitoring	Analyses of Exosomes in the Cerebrospinal Fluid for Breast Cancer Patients With Suspicion of Leptomeningeal Metastasis	N/A	Breast cancer; leptomeningeal metastasis	Interventional	Withdrawn
NCT04288141	Predictive/response biomarker	HERdi PREDICT: A Pilot Study to Measure the Expression of the HER2-HER3 Dimer in Samples From Patients With HER2-Positive Breast Cancer Receiving HER2-Targeted Therapies	N/A	HER2-positive breast cancer	Observational	Unknown
NCT02662621	Diagnostic biomarker	Pilot Study With the Aim to Quantify a Stress Protein in the Blood and in the Urine for Early Diagnosis of Malignant Solid Tumors (EXODIAG)	N/A	Breast, ovarian, and lung cancer	Interventional	Completed
NCT07574749	Molecular subtyping/predictive application	Molecular Subtyping of Breast Cancer-Derived Small Extracellular Vesicles (sEVs) to Predict Therapeutic Efficacy	Phase II	Breast cancer	Observational	Recruiting

To facilitate comparison of the therapeutic performance of engineered exosome- and EV-based platforms, representative preclinical studies in breast cancer immunotherapy are summarized in Table 3. The table highlights the vesicle source, engineering strategy, therapeutic cargo or immunomodulatory component, breast cancer model, targeting or mechanism of action, and key therapeutic outcomes reported in each study.

**Table 3:** Representative engineered exosome- and extracellular vesicle-based therapeutic platforms evaluated in breast cancer models.

Study	Vesicle source/platform	Engineering strategy/targeting	Therapeutic cargo or immune function	Breast cancer model	Key performance parameters/major outcomes
Ohno et al. [155]	HEK293T-derived EVs	PDGFR transmembrane domain fused with GE11 peptide for EGFR targeting	let-7a miRNA	EGFR-expressing breast cancer models	Targeted EVs enhanced delivery of let-7a and suppressed tumor development in vitro and in vivo.
Tian et al. [144]	Immature dendritic-cell-derived exosomes	Lamp2b–iRGD surface engineering for αv-integrin targeting	Doxorubicin (DOX)	MDA-MB-231 and MCF-7; mouse xenograft	Approximately 20% DOX encapsulation efficiency; enhanced tumor targeting and antitumor activity with a favorable toxicity profile.
Limoni et al. [156]	HEK293T-derived exosomes	LAMP2b–DARPin G3 targeting HER2	TPD52-targeting siRNA	HER2-positive SKBR3 cells	Specific binding and uptake by HER2-positive cells with successful siRNA delivery and TPD52 silencing.
O'Brien et al. [130]	MSC-derived EVs	Genetic enrichment of therapeutic miRNA	miR-379	Breast cancer cells and mouse models	miR-379 delivery reduced COX-2 expression and produced a significant antitumor effect in vivo.
Gomari et al. [145]	MSC-derived exosomes	LAMP2b–DARPin targeting HER2	DOX	HER2-positive breast cancer models	Targeted exosomes showed greater binding to HER2-positive cells; DOX-loaded targeted vesicles produced stronger cytotoxicity than free DOX under the reported conditions.
Yang et al. [157]	MSLN-targeted CAR-T-cell-derived exosomes	Genetic expression of anti-MSLN CAR	CAR-associated cytotoxic machinery, including perforin and granzyme B	MSLN-positive TNBC; in vivo xenografts	Significant inhibition of MSLN-positive TNBC growth; dose-dependent antitumor activity with no obvious systemic toxicity reported.
Yildirim et al. [140]	4T1 breast cancer-derived exosomes	Exogenous loading with TLR agonists	K-type CpG ODN + poly(I:C)	4T1 mammary tumor-bearing mice	Enhanced antigen-specific cellular and memory T-cell responses and reduced established tumor growth in vivo.
Ji et al. [158]	DC2.4-derived exosomes	Surface display of CD62L and OX40L	Immune-amplifying exosomal surface proteins	4T1 syngeneic breast cancer model	Strong tumor-draining lymph-node homing; increased effector T cells, reduced Treg induction, and inhibition of tumor development.
Peng et al. [146]	RBC-derived EVs	Engineered EV-mediated delivery	RIG-I agonists	Human and murine breast cancer models	Robust activation of RIG-I signaling, induction of antitumor immune responses, and tumor-cell apoptosis; demonstrated feasibility of EV-mediated innate immune activation.
Huang et al. [22]	α-lactalbumin-engineered breast cancer-derived exosomes (HELA-Exos)	α-LA surface engineering + electroporation	ELANE + Hiltonol (TLR3 agonist)	MDA-MB-231/TNBC; patient-derived breast cancer organoids	Induced immunogenic cell death, activated cDC1s, promoted tumor-reactive CD8^+^ T-cell cross-priming, and produced stronger tumor inhibition than free Hiltonol.
Hu et al. [139]	Engineered EV-like nanovesicles (eNVs-FAP)	Surface/functional engineering to deliver FAP	FAP-based tumor-vaccine strategy	Breast cancer and other tumor models	Induced CTL responses and inhibited tumor growth; breast cancer was among the evaluated tumor models.
Cheng et al. [159]	Expi293F-derived engineered exosomes (GEMINI-Exos)	Surface display of anti-CD3, anti-EGFR, PD-1 and OX40L	T-cell redirection + checkpoint blockade + costimulation	EGFR-positive TNBC	Increased T-cell recruitment/activation, enhanced CD8^+^ T-cell infiltration, reduced Treg-mediated suppression, and strongly inhibited established TNBC tumors in mice.
Wang et al. [152]	Engineered EVs	Anti-HER2 scFv surface targeting	HChrR6-encoding mRNA + CNOB prodrug	HER2-positive BT474 xenografts	Specific HER2 targeting and localized prodrug activation; EXO-DEPT markedly suppressed growth of HER2-positive xenografts.
Tao et al. [150]	CAR-NK-cell-derived exosomes	CAR expression + T7 peptide modification; integration with micelle nanobomb	ROS-amplified photodynamic/ferroptosis-related therapeutic cargo	HER2-positive breast cancer brain-metastasis model	Crossed the BBB, selectively targeted HER2^+^ tumor cells, significantly suppressed tumor growth, and prolonged survival without evident systemic toxicity.
Yu et al. [160]	M1-like macrophage-derived exosomes	OX40L overexpression on exosomal surface	OX40/OX40L immune activation	Mouse breast cancer model	Activated adaptive immunity, reprogrammed M2→M1 TAMs, enhanced innate/adaptive immune synergy, and significantly inhibited tumor growth and metastasis. (PubMed)
Aare et al. [74]	Genetically engineered exosomes	Surface expression of anti-PD-L1; STAT3-siRNA loading	STAT3 siRNA + paclitaxel combination	Doxorubicin-resistant MDA-MB-231 TNBC; 3D spheroids and xenografts	Marked reduction in tumor burden (P < 0.001), suppression of PD-L1/STAT3-related signaling, enhanced apoptosis, and time-dependent tumor accumulation; no evident off-target toxicity in MCF-10A cells.
Parvin et al. [161]	HEK293-derived engineered exosomes	Surface display of CD206-targeting peptide (CSPGAKVRC) + Fc-mIgG2b	Selective targeting and ADCC-mediated depletion of CD206^+^ M2 macrophages	4T1/BALB/c and AT3/C57BL/6 syngeneic breast cancer models	Selective M2 macrophage depletion without significant lung/liver retention or organ toxicity; delayed tumor recurrence and metastasis and improved survival (P < 0.02).

## 7 Challenges and future perspectives

Despite significant advances in the development of engineered exosome-based and EV-based platforms for breast cancer immunotherapy, several challenges must be addressed before these nanoscale systems can be successfully translated into clinical practice. One of the primary limitations is the lack of standardized protocols for EV isolation, purification, and characterization, particularly for exosome-enriched preparations. Current methods, such as ultracentrifugation and size-exclusion chromatography, often result in heterogeneous populations, low yield, and potential contamination with other EVs, thus compromising reproducibility and scalability [162]. The MISEV2023 recommendations provide an updated consensus framework for extracellular vesicle nomenclature, collection and preprocessing, separation, characterization, functional assessment, and in vivo studies, and emphasize the need for transparent reporting and appropriate methodological controls [23]. Application of these recommendations to engineered EV and exosome-based research could improve inter-study comparability and facilitate the establishment of reproducible quality-control criteria.

Another critical challenge lies in the efficient and reproducible loading of therapeutic cargoes into EVs, including exosome-enriched vesicles. Although techniques such as electroporation, sonication, and transfection have been employed, each comes with inherent limitations regarding cargo stability, loading efficiency, and potential vesicle damage [163]. Moreover, targeting specificity remains a major concern. While surface modification strategies using ligands, peptides, or antibodies have shown promise in enhancing tumor targeting, off-target effects and immune clearance still pose substantial barriers [164]. Standardized characterization of engineered EV preparations should therefore include assessment of particle size and concentration, morphology, surface markers, cargo identity and abundance, purity, biological activity, and potential contaminants, consistent with the principles outlined in MISEV2023 [23].

Immunogenicity and safety profiles of engineered EV-based therapeutics must also be carefully evaluated. Although EVs, including exosome preparations, exhibit lower immunogenicity compared with many synthetic nanocarriers, their source, surface composition, and cargo can influence host immune responses. Long-term biodistribution, clearance mechanisms, potential off-target accumulation, and the possibility of horizontal gene transfer require comprehensive in vivo studies to establish therapeutic safety [165]. MISEV2023 also emphasizes appropriate approaches for studying extracellular vesicle release, uptake, function, and in vivo fate, highlighting the importance of rigorous experimental controls when interpreting biodistribution and functional data [23].

Regulatory and manufacturing hurdles further complicate clinical translation. Exosome-based products and other EV-derived therapeutics are biologically derived and structurally complex, making it challenging to establish quality-control criteria, batch-to-batch consistency, potency assays, and cost-effective large-scale production. Regulatory oversight is particularly important because EV-based products intended to treat human diseases are subject to regulatory requirements. In the United States, the U.S. Food and Drug Administration (FDA) states that exosome products intended to treat diseases or medical conditions in humans require FDA approval, and no exosome product is currently FDA-approved [166]. Therefore, clinical development requires rigorous demonstration of product identity, quality, safety, and efficacy, together with appropriate manufacturing and clinical-investigation procedures. Similar principles of product characterization, manufacturing consistency, quality assurance, and evidence of safety and efficacy are essential when considering regulatory pathways in other jurisdictions, including the European Union. Accordingly, harmonization of manufacturing and characterization procedures and development of Good Manufacturing Practice (GMP)-compliant production platforms remain critical priorities [166,167].

Looking forward, future research should focus on developing scalable and reproducible methods for EV production, exosome enrichment, and functionalization, enhancing targeting accuracy, and integrating multi-omic tools to engineer personalized EV-based therapies. Furthermore, combining engineered EV platforms with other immunotherapeutic modalities, such as immune checkpoint inhibitors or cancer vaccines, may yield synergistic effects and improve clinical outcomes. Integration of standardized MISEV2023-informed characterization with advanced in vivo tracking, quantitative potency assays, and GMP-compatible manufacturing could further facilitate the transition from preclinical studies to clinical trials [23]. Ultimately, interdisciplinary collaboration between bioengineers, immunologists, oncologists, and regulatory authorities will be crucial to unlocking the full translational potential of engineered exosome and EV-based platforms in breast cancer immunotherapy.

## 8 Conclusion

Engineered exosomes have emerged as a transformative platform in the landscape of breast cancer immunotherapy, offering a biocompatible, versatile, and cell-targeted delivery system for a wide range of therapeutic agents, including proteins, nucleic acids, and small molecules. As a specialized subset of extracellular vesicles, exosomes provide unique biological features; however, many engineered vesicle systems currently investigated in cancer therapy represent heterogeneous EV-based preparations rather than experimentally confirmed exosomes. Their intrinsic ability to evade immune surveillance, navigate biological barriers, and selectively interact with recipient cells positions them as a promising alternative to traditional nanocarriers. Moreover, advances in surface engineering and cargo loading strategies have enhanced the precision and therapeutic efficacy of engineered exosome and EV-based systems, enabling their application in targeted tumor suppression and immune modulation.

Despite these encouraging developments, the clinical translation of engineered exosome-based and EV-based therapeutics remains in its early stages. Key challenges including manufacturing scalability, purification standardization, targeting specificity, and safety validation must be addressed through rigorous research and technological innovation. Nevertheless, the growing body of preclinical evidence supports the feasibility and potential of engineered EV platforms, including exosome-based immunotherapies, in breast cancer treatment.

As the field progresses, integration of multi-omics data, artificial intelligence-driven design, and personalized medicine approaches will likely play pivotal roles in optimizing EV engineering and therapeutic delivery. With continued interdisciplinary collaboration and support from regulatory bodies, engineered exosome and EV-based platforms hold the promise to redefine the paradigm of breast cancer immunotherapy and contribute to more effective, targeted, and patient-centric cancer care.

## CRediT authorship contribution statement

**Niloufar Orooji:** Writing – original draft, Investigation, Conceptualization. **Mahsa Ghasemian-Irani:** Writing – original draft, Investigation. **Nasrin Salari:** Writing – original draft, Investigation. **Tamana Eskandari:** Writing – original draft, Investigation. **Mehrshad Shams:** Writing – original draft, Investigation. **Fatemeh Tavassoli Razavi:** Writing – original draft, Visualization, Investigation. **Dariush Haghmorad:** Writing – review & editing, Writing – original draft, Validation, Supervision, Investigation, Conceptualization.

## Declarations

We confirm that no artificial intelligence (AI) tools were used to generate or write the scientific content of this manuscript. Therefore, no AI declaration is required according to the journal's policy.

## Funding

This research received no external funding.

## Declaration of competing interest

The authors declare that they have no known competing financial interests or personal relationships that could have appeared to influence the work reported in this paper.

## Data Availability

No data was used for the research described in the article.
